# Focus Issue: Neck Dissection for Oropharyngeal Squamous Cell Carcinoma

**DOI:** 10.5402/2012/547017

**Published:** 2012-01-15

**Authors:** Kathryn M. Van Abel, Eric J. Moore

**Affiliations:** Department of Otolaryngology, Head and Neck Surgery, Mayo Clinic, Rochester, MN 55905, USA

## Abstract

The staging and prognosis of oropharyngeal squamous cell carcinoma is intimately tied to the status of the cervical lymph nodes. Due to the high risk for occult nodal disease, most clinicians recommend treating the neck for these primary tumors. While there are many modalities available, surgical resection of nodal disease offers both a therapeutic and a diagnostic intervention. We review the relevant anatomy, nodal drainage patterns, clinical workup, surgical management and common complications associated with neck dissection for oropharyngeal squamous cell carcinoma.

## 1. Introduction

Each year, 5000 new cases of oropharyngeal cancer are diagnosed in the US, and 85–90% of these are confirmed as squamous cell carcinoma (SCC) [1]. Cervical lymph node status remains the most important prognosticator in head and neck squamous cell carcinoma (HNSCC) in the absence of distant metastases, reducing 5-year survival by 50% [2, 3]. While the choice of management for occult metastases is complex, most clinicians agree that treatment should be chosen over observation when the risk of occult disease is 20% or greater [4]. The incidence of occult metastases in clinically node-negative necks (cN0) in OPSCC has been reported to be greater than 30% in some series [5, 6]. The importance of assessing and managing the cervical nodal basin in OPSCC is therefore of utmost importance and is the focus of the current paper.

## 2. Anatomy and Lymphatic Drainage of the Oropharynx

Prognosis for patients with OPSCC is closely associated with the involvement of cervical lymph nodes. Therefore, an understanding of the anatomical subsites and lymphatic drainage patterns of each is crucial. The oropharynx is bounded by the posterior edge of the hard palate superiorly, the pharyngeal wall posteriorly, the tonsillar complexes (including the anterior and posterior tonsillar pillars, true tonsil, and tonsillar fossa) laterally, the circumvallate papillae and palatoglossal muscles anteroinferiorly, and the vallecula and hyoid bone inferiorly. The surgical anatomy of this area is classically divided into four distinct subsites: (1) base of tongue (BOT), (2) soft palate, (3) tonsillar complex, and (4) posterior pharyngeal wall (PPW) [7]. These subsites are independently important, and as Lindberg stated in his classic work on lymphatic drainage patterns in the head and neck, “metastases from primary lesions of the oropharynx have some common locales [8].” A thorough understanding of these drainage patterns is a prerequisite to the surgical neck dissection (ND) for OPSCC.

### 2.1. Base of Tongue

The BOT can be defined anteriorly by the circumvallate papillae, laterally by the glossopalatine sulci, and inferiorly by the vallecula [9]. This area includes the pharyngoepiglottic folds as well as the glossoepiglottic fold [10]. The lymphatics of the BOT drain primarily to the upper two thirds of the jugular lymphatic chain, often bilaterally [7]. Hollinshead stated that the lymphatics posterior to the vallate papillae drained to the nodes of the upper part of the deep cervical chain, with a predilection for the jugulodigastric node [11]. Lindberg associated the midline position of the BOT with the frequency of bilateral cervical node involvement [8]. In his report, bilateral subdigastric node involvement was more common than midjugular node involvement, posterior cervical nodal disease was uncommon, and low jugular or supraclavicular nodes were rare [8].

### 2.2. Soft Palate

The soft palate is defined anteriorly by the hard palate, laterally by the palatopharyngeal and superior pharyngeal constrictor muscles, and posteriorly by the palatopharyngeal arch and uvula. The lymphatics of the soft palate have three distinct systems, which drain (1) medially to the middle third of the jugular chain, (2) laterally to the retropharyngeal (RP) lymphatics, and (3) anteriorly to the hard palate and subsequently into the submental and submandibular nodal groups [7]. The lymphatics in the uvula drain primarily into the upper jugular chain, while the vessels draining the upper or posterior surface of the soft palate drain laterally via the pharyngeal lymphatics to end in the RP nodes [11]. Lindberg found that, as a midline structure, the incidence of bilateral metastases in OPSCC of the soft palate was high, with the jugular nodes the most frequently involved [8].

### 2.3. Posterior Pharyngeal Wall

The PPW spans the area defined by the soft palate, the epiglottis, the borders of the tonsillar complexes, and the lateral aspects of the piriform sinuses inferiorly [9]. The lymphatic drainage from the PPW is primarily via the jugular chain bilaterally to the upper jugular nodes in the subdigastric group [8]. The midjugular group is also frequently involved, as is the posterior cervical triangle, while supraclavicular disease is rare [8].

### 2.4. Tonsillar Complex

The tonsillar complex is composed of the anterior and posterior tonsillar pillars, the true palatine tonsil, and the tonsillar fossa. Primary tumors of the tonsillar complex frequently metastasize to the lateral RP nodes and the upper third of the ipsilateral jugular lymphatic chain, with a smaller proportion draining to the middle third of the jugular lymphatic chain [7]. Lindberg found that the tonsillar node, within the subdigastric group, was always involved first in cervical metastasis [8]. He also found that both mid and low jugular nodes were frequently involved, and metastases within the posterior cervical triangle were not uncommon [8].

### 2.5. Potential Spaces

When considering oncology of the oropharynx, it is also important to understand the association of two relevant potential spaces. The RP space is located posterior to the oropharynx behind the pharyngeal constrictors. Invasion into this space by OPSCC significantly increases the risk of bilateral regional metastases [1]. RP nodes are most commonly involved in PPW OPSCC. Ballantyne reported positive RP nodes in 44% of patients with OPSCC in this subsite [12]. Hasegawa and Matsuura reviewed 11 cases of stage III/IV OPSCC and concluded that carcinoma of the oropharynx drains directly to the RP nodes, and therefore the evaluation of the RP lymph nodes is critical in the assessment of SCC of the pharynx [13]. The second space, the parapharyngeal space, is important when considering lateral spreading tumors. This space is often described as an inverted pyramid bounded by the skull base, lateral pharyngeal constrictors, and hyoid cornu [14].

## 3. Nodal Classification

Despite a careful understanding of the anatomy and lymphatic drainage patterns of subsites within the oropharynx, there is a wide variety of individual anatomy and incidence of cervical nodal involvement. A careful understanding of the classification system presented by the Memorial-Sloan Kettering Group [15] and further modified by the American Head and Neck Society's Neck Dissection Committee [16] is therefore warranted. This classification system contains six levels, with level I, II, and V divided into two subgroups designated either A or B and is based largely on the biologic significance of positive nodes [7]. Each level has a general name describing the group of lymph nodes within its boundaries and defined borders based on anatomic, radiologic, and surgical landmarks [17]. We will focus our review on the surgical landmarks defining each level. The frequency of nodal involvement in OPSCC based on this classification system is reviewed in Table 1. 

The submental lymph nodes make up level IA. They can be found within the submental triangle, which is bounded laterally by the anterior bellies of the digastric muscles, inferiorly by the hyoid, and superiorly by the mandibular symphysis [17]. The floor of this space is formed by the mylohyoid muscle. The submandibular lymph nodes are found in level IB as defined surgically by the body of the mandible superiorly, the digastric tendon attachment to the hyoid bone inferiorly, the anterior belly of the digastric muscle anteriorly, and by the posterior edge of the submandibular gland posteriorly [17]. 

Level II defines the superior jugular lymph nodes and is divided into Level IIA and IIB by the spinal accessory nerve. Level IIA is defined superiorly by the skull base, inferiorly by the carotid bifurcation (or the inferior border of the hyoid bone), posterolaterally by a vertical plane defined by the spinal accessory nerve (SAN), and anteriorly by the posterior border of the submandibular gland [17]. Level IIB is referred to as the submuscular recess or posterior triangle apex. It is defined superiorly by the skull base, inferiorly by the carotid bifurcation (or the inferior border of the hyoid bone), laterally by the sternocleidomastoid muscle, and anteriorly or medially by the vertical plane defined by the path of the SAN [17]. Its floor is defined by the splenius capitus muscle [18]. 

The midjugular lymphatic group, or level III, is defined superiorly by the carotid bifurcation, inferiorly by the omohyoid muscle, laterally by the sensory branches of the cervical plexus, and medially by the sternohyoid muscle [17]. 

Level IV is described as the inferior jugular nodes and is defined superiorly by the omohyoid muscle, inferiorly by the clavicle, laterally by the sensory branches of the cervical plexus of nerves or the lateral border of the sternocleidomastoid muscle, and medially by the sternohyoid muscle [17]. 

Level V is again subdivided into level VA and VB. It defines the posterior triangle lymph node group and is variably involved in OPSCC. Level VA is defined superiorly by the junction of the SCM and the trapezius muscle, inferiorly by the horizontal plane defined by the inferior border of the cricoid cartilage, laterally by the anterior border of the trapezius muscle, and medially by the sensory branches of the cervical nerve plexus [17]. Level VB is defined superiorly by the horizontal plane of the inferior border of the cricoid cartilage, inferiorly by the clavicle, laterally by the anterior border of the trapezius, and medially by the sensory branches of the cervical plexus [17]. 

Level VI, known as the anterior or central neck compartment, is important to define for completeness but is rarely involved in OPSCC [17]. It is defined superiorly by the hyoid bone, inferiorly by the superior edge of the manubrium of the sternum, and bilaterally by the common carotid arteries [19].

## 4. Diagnostic Evaluation of the Neck in OPSCC

Staging the neck using the nodal levels described above, as well as the TNM system recommended by the American Joint Commission on Cancer (AJCC) (Table 2), is a prerequisite for surgical management of the neck in OPSCC [20]. The National Comprehensive Cancer Network (NCCN) includes the following in their 2011 guidelines for the evaluation of the neck in OPSCC: history and physical, biopsy, HPV testing for prognosis (suggested), chest imaging, computed tomography (CT) with contrast and/or magnetic resonance imaging (MRI) of both the primary site and neck, 18-fluorodeoxyglucose positron emission tomography and computed tomography (PET-CT) for stage III–IV (consideration), dental evaluation as indicated, and examination under anesthesia with endoscopy as clinically indicated [21]. 

Physical examination and palpation of the neck has a relatively low sensitivity and specificity for diagnosing lymphatic metastasis [23]. McGuirt et al. reported the sensitivity of palpation alone to be 70% [24]. The addition of CT scans to clinical findings increases diagnostic detection to 80% with an accuracy of 70–80% [25]. According to Cohan et al., MRI offers superior soft tissue contrast, increased resolution of bone marrow involvement, and improved resolution of perineural spread compared to CT [14]. Shingaki et al. concluded that the addition of CT, MRI, and biopsy to palpation increases the diagnostic value for cervical disease [26]. A relatively new addition to the diagnostic armamentarium is PET-CT. This modality has been shown to assist in the diagnosis and detection of regional and distant metastases in HNSCC [27, 28]. PET-CT has an average sensitivity of 87–90% and specificity of 80–93% [29, 30]. This tool can be used to guide dissection of high-risk areas outside the bounds of the routine ND [31]. However, PET-CT is limited by a relatively low resolution (4-5 mm), high false positive rate, cost, and interobserver variability [32]. Despite advances in technology, the gold standard for staging the neck remains histopathologic tissue analysis. Rodrigo et al. note that even the most recent techniques of CT, MRI, ultrasonography, PET-CT, and ultrasound guided FNA have reached a sensitivity of no more than 80–85% [33]. The primary limitation of these studies appears to be the inability to detect micrometastases [34].

Pathology of the neck specimens provides information regarding size of metastases, number of involved nodes, presence of extracapsular spread (ECS), additional tumor deposits, and micrometastases [2]. However, despite the routine use of pathologic staging and our dependence on it as the gold standard for staging the neck, Shah and Gil. report the risk of recurrence in the pathologically node-negative (pN0) neck to be as high as 10% [35]. This is relevant in that the diagnosis of a false negative pN0 is associated with a poor prognosis [36]. This has spurned investigation into the use of molecular markers which might improve diagnostic accuracy. Using reverse transcriptase polymerase chain reaction (RT-PCR), Seethala found that 20–30% of pN0 necks by light microscopy and immunohistochemistry (IHC) were “molecular positive” [2]. This technique is limited by a high false positive rate, the requirement of frozen specimens, the loss of morphologic comparison, and the lack of a defined or consistent “threshold” for positivity [2]. Some authors hypothesize that further understanding of micrometastases, isolated tumor cells, and molecular characteristics may help to explain a subset of the 10% of regional recurrences in the neck despite a pN0 designation based on light microscopy and IHC [2]. In addition, further research into the use of additional markers and the use of a multiplex approach to diagnosis could help explain regional failures not explained by surgical failure and nonlymphatic spread of disease [2, 37].

The inability of routine pathologic analysis to detect 100% of tumor and the associated morbidity associated with routine ND has led to investigation into the use of sentinel lymph node biopsy (SLN) for staging the neck in OPSCC. Ferlito et al. suggest that since only 25–30% of cN0 necks are upstaged to pN+ necks following ND, the majority of elective surgical management has no therapeutic benefit other than to confirm clinical staging [38]. Furthermore, Burcia et al. suggest that while SLN sampling allows the pathologist to focus their efforts on a smaller tissue sample, routine staging methods requiring evaluation of large neck specimens leads to a high rate of false negatives and may underestimate the number of invaded lymph nodes per patient [39]. The Second International Conference on Sentinel Node Biopsy In Mucosal Head and Neck Cancer suggested that SLN biopsy can be used in the following settings: (1) staging of the ipsilateral neck in unilateral cT1/T2 cN0 tumors, (2) staging of the ipsilateral and contralateral neck in cT1/T2 cN0 midline tumors or tumors crossing the midline, and (3) staging of the contralateral neck in cT1/T2 cN+ (ipsilateral neck) midline tumors or tumors crossing the midline [40]. Regardless of the potential for this staging modality, its routine use in the management of the neck in OPSCC is still inadequately defined.

## 5. Neck Dissection

### 5.1. Past and Present History

A discussion regarding surgical management of the neck in OPSCC cannot be undertaken without first reviewing its history. The importance of nodal involvement in head and neck cancers was first reported in the medical literature in the 18th century and was considered a sign of incurable disease until the mid-1800s [41]. In 1880, Kocher presented the first description of removing cervical nodes in the submandibular triangle to access a cancer of the tongue [42]. The first documented ND was performed in 1888 by Jawdynski [43], a polish surgeon, and the first detailed description of the radical neck dissection (RND) was published by Crile in 1906 [44]. Interestingly, while 36 of the 132 RNDs described by Crile were en bloc procedures, 96 demonstrated more selective surgical techniques [44]. Crile recommended preserving the internal jugular vein (IJV) and sternocleidomastoid muscle (SCM) in a cN0 neck and advised dissection of only the lymphatic basin draining the primary tumor if there was no gross disease [44].

Despite the forward thinking of Crile, the en bloc resection remained the mainstay for surgical management of the neck until the mid-1900s. By the early 1950s, Dr. Hayes Martin and colleagues had performed over 190 NDs, and, as a staunch defendant of the RND, he disagreed with any suggestion that the RND be modified [45]. However, the significant morbidity associated with the RND, especially related to postoperative shoulder dysfunction, provided an impetus for research into alternate approaches to the neck. The discovery that lymphatic structures within fascial compartments of the neck could be removed without sacrificing nonlymphatic structures [7, 46] prompted investigation into more conservative techniques. Ward and Robben reported the first form of a modified radical neck dissection (MRND) by in which they spared the spinal accessory nerve (SAN) [46]. This procedure resulted in decreased shoulder morbidity without compromising oncologic outcome [17]. MRND was popularized by Bocca, who formally defined it as the removal of the lymphatic tissue in levels I–V, with preservation of at least one of the nonlymphatic structures classically included in RND: IJV, SAN, or SCM [47]. It was not until the 1960s that authors began reporting preservation of select lymph node groups, which marked the first formal introduction of the selective neck dissection (SND) [48]. These advances were supported by the finding that metastases from the head and neck tend to follow fairly constant and predictable pathways, which allows the surgeon to tailor surgical dissection to target the areas most at risk [8, 49]. Furthermore, in the 1980s, studies by the Brazilian Head and Neck Cancer Group found that SND was equivalent to MRND for the cN0 neck in regards to neck recurrence rates and long-term survival [50]. 

As the procedures available for surgical management of the neck evolved, authors produced a myriad of confusing and nonsystematic terminology. In 1988, the Committee for Head and Neck Surgery and Oncology of the American Academy of Otolaryngology-Head and Neck Surgery called together a task force in order to simplify terminology, define procedures and surgical structures, and classify cervical metastases based on biology and the principles of surgical oncology [17]. The outcomes from this meeting with the recent modifications made by the Committee for Neck Dissection Classification of the American Head and Neck Society [51] have resulted in the current classification system used to describe NDs in the modern era.

### 5.2. Definitions

The most basic terms used to describe NDs are defined by surgical intent. The *therapeutic ND* is performed for a cN+ neck, while an *elective ND* is described for cN0 necks at high risk for occult metastases [7]. In addition, a *staged ND* often refers to a planned ND following primary radiation or chemoradiation therapy while a *salvage ND* refers to an ND performed to remove persistent (*early salvage ND*) or recurrent (*late salvage ND*) nodal disease following nonoperative therapy [7]. 

The American Head and Neck Society Committee for Neck Dissection Classification described four major categories of ND currently available based on the anatomic structures resected (Table 3) [51]. These include the radical, modified, selective, and extended NDs. 

The RND, as first described by Crile in 1906, is the comprehensive standard to which all other dissections are compared. In this procedure, the SAN, IJV, and SCM are resected in addition to all lymphatic tissue in levels I–V. Currently, RND is indicated for patients who have cN+ disease demonstrating ECS with extension to involve the SAN, IJV, and/or the SCM [17]. 

The MRND as described initially by Ward and Robben [46] and further defined by Medina [52] involves en bloc resection of lymphatic tissue contained in level I–V [7] with the preservation of one or more nonlymphatic structures (SAN, IJV, or SCM) [7]. The description of a MRND can be subclassified as type I–III depending on the nonlymphatic structure(s) removed during resection (Table 3) [52]. This dissection is indicated for cN+ disease with no evidence of extension into the nonlymphatic structures, especially when multilevel disease is present [7]. 

The SND has been found to offer an oncologically safe surgery while decreasing overall morbidity and increasing functional and cosmetic outcomes for the appropriately selected patient population [7]. During this dissection, the surgeon targets lymph node levels at risk for occult metastases. This dissection preserves the nonlymphatic structures and allows the surgeon to tailor the dissection to the primary tumor and its lymphatic drainage patterns. Because SND refers to preservation of at least one of the five neck levels included in the classical RND, there is significant room for variability. Therefore, authors have defined various terms to describe different techniques, such as the supraomohyoid SND (levels I–III), lateral SND (level I–IV), posterolateral SND (level II–V), and the anterior or central SND (level VI) [2]. While these terms are descriptive in theory, they do leave room for misinterpretation. Therefore, the Committee for Head and Neck Surgery and Oncology of the American Academy of Otolaryngology—Head and Neck Surgery updated the ND classification in 2002 and recommended that each SND should be listed as SND with each variant depicted with brackets to denote the levels or sublevels removed [16, 53]. 

Finally, the extended ND is employed when there is a high risk or clinical suspicion for metastatic disease outside of the classic lymph node levels and may be applied to any of the previously described NDs [17]. Areas targeted by extended ND may include additional lymph node basins such as the periparotid, retropharyngeal, parapharyngeal, superior mediastinal, postauricular, suboccipital, or buccinators [17]. This type of dissection may also target such nonlymphatic structures as muscle, vasculature, or nerves when at risk or involved by tumor [17].

### 5.3. Level IIB and V in OPSCC

The motivation behind developing more selective ND techniques has been driven in part by the desire to decrease associated morbidity without sacrificing oncologic surgical principles and outcomes. The most significant sequelae associated with ND is shoulder dysfunction and is an important consideration in treatment of the neck for OPSCC. Taylor et al. looked at quality of life indicators via the Neck Dissection Impairment Score and found that the most important variables included age, weight, radiation treatment, and ND type [54]. Injury of the SAN can be related to excess traction or elevation [18] and is primarily associated with dissection of levels IIB and V.

RND was classically known to cause denervation of the trapezius muscle leding to a syndrome of pain, weakness, and deformity of the shoulder girdle [7]. While Leipzig et al. [55] showed that any form of ND can result in shoulder dysfunction, they reported it to occur more frequently when the SAN is worked around, skeletonized, or resected. Sobol et al. compared RND with MRND and predictably found that patients undergoing RND had significantly worse shoulder outcomes [56]. However, MRND still resulted in associated shoulder morbidity [56], and the combination of this with the finding that SND offered an oncologically safe outcome in select patients and pushed many surgeons to evaluate the efficacy of this method. Sobol et al. further demonstrated that patients undergoing SND (level I–III) had significantly less shoulder dysfunction at 16 weeks postoperatively than MRND or RND [56]. Chepeha et al. demonstrated a worse Constant's Shoulder Score for MRND than SND in 32 NDs of each type [57]. Based on these and similar studies, the SND offers the best chance for preserving shoulder function and avoiding pain syndromes when the surgeon is able to avoid dissecting level IIB and V.

Preservation of level IIB in OPSCC is a controversial topic, as the landmark studies on cervical nodal disease patterns for OPSCC reported a very high incidence of metastatic disease in level II. However, these studies often did not evaluate the difference between disease in level IIa and IIB. Injury to the SAN during dissection of level IIB can be related to traction, elevation, skeletonization, or ischemia due to ligation of the occipital artery [18]. The data on involvement of IIB nodes for all subsites in HNSCC ranges from 0–5.6% for cN0 cases, and 0–16.7% for cN+ disease [18]. Lee et al. specifically analyzed level IIB involvement in OPSCC and found that 16.7% of ipsilateral and 8.3% of contralateral level IIB nodal basins were pathologically positive in cN+ disease [18]. In the cN0 neck, 0/36 necks (21 ipsilateral, 15 contralateral) demonstrated disease in level IIB [18]. Based on these results, they recommended preservation of level IIB for cN0 OPSCC [18]. Weigand et al. reviewed 77 NDs for OPSCC and also found that, in the cN0 neck, no patient was found to have occult Level IIB disease while 25.6% of cN+ patients had disease in this area [58]. Several other authors have reported an increased risk for occult IIB disease in patients demonstrating multiple nodal involvement, most commonly associated with level II and III disease, as well as level IIa involvement, high nodal stage, or ECS [18, 59, 60]. Therefore, while preservation of level IIB should be strongly considered, risk factors for occult metastases should be thoroughly evaluated in an effort to maintain oncologic safety.

Preservation of level V in cN0 OPSCC is less controversial, as very few studies have suggested significant involvement in this region. In a study of 51 RNDs in 1976, Skolnik et al. found no metastases to the posterior triangle of the neck regardless of primary site (larynx, pharynx, and oral cavity) or the status of the jugulodigastric lymph nodes [61]. In one of the largest retrospective reviews on the subject, Shah evaluated 1119 RND specimen and found that primary tumors of the oropharynx predominantly metastasize along the jugular lymphatic chain (Levels II, III, and IV) [62]. In addition, regardless of subsite, when levels I–IV were negative for occult disease, the posterior cervical triangle was never involved [62]. This supports Lindberg's finding that in the absence of metastases in levels I and II, involvement of the low jugular and posterior triangle nodes is exceedingly rare [8]. Candela et al. also found that while the jugular chain (level II-III) was the most commonly involved in OPSCC, nodal involvement in the posterior cervical triangle nodes was almost always associated with disease at other levels [63]. They found that only 6% of OPSCC primaries were associated with posterior cervical triangle disease (level V) [63]. As detailed above, the incidence of metastatic disease in level V for OPSCC is relatively low, and therefore there is a general consensus that if a patient presents with a cN0 neck or a cN+ neck with no evidence of level V disease and without multilevel involvement, a SND can be safely performed preserving level V and avoiding dissection around the SAN in this area.

### 5.4. Surgical Management of the Neck in OPSCC

The choice of dissection technique for surgical management of the neck depends on the primary tumor, the clinical status of the neck, and ultimately on the pathologic status of the neck. While consideration of primary radiation, chemoradiation therapy, or observation is beyond the scope of this review, it is important to remember that management of the neck is often driven by management the primary tumor for early staged tumors while multimodal therapy is advocated for more advanced OPSCC tumors [14, 22].

Understanding the predilection for bilateral cervical involvement helps guide both evaluation and choice of ND procedure. Up to 20% of patients with T1 or T2 BOT OPSCC will have bilateral nodal disease at presentation [14]. In patients with palatal OPSCC staged T1 or T2 up to 20% present with cN+ disease, 60–70% of T3 and T4 palatal disease present with regional metastasis [14], and up to 50% may present with bilateral nodal disease [14, 64]. OPSCC tumors of the PPW often cross midline resulting in a relatively high risk for bilateral nodal involvement. In addition, 66–76% of patients with tonsillar OPSCC present with clinically positive nodal disease, most commonly in the jugulodigastric nodal group [1, 64, 65]. In tumors that involve the true tonsil as well as the posterior tonsillar pillar, up to 22% have been reported to present with bilateral nodal involvement, whereas primary tumor growth in the anterior pillar alone is associated with a 6% risk of bilateral neck disease [1, 64, 65]. Contralateral necks with evidence of metastatic spread, or at high risk of metastatic spread, must be considered for dissection if the treatment plan is operative. 

The National Comprehensive Cancer Network: Head and Neck Cancer Guidelines [21] have detailed strategies for the management of OPSCC. For an N0-1 neck associated with a T1–4 OPSCC primary, the surgical treatment arm recommends an ipsilateral or bilateral ND as indicated based on primary tumor risk factors for bilateral involvement and diagnostic workup [21]. Additional adjuvant chemo- or radiation therapy is guided by adverse features identified intraoperatively, which include ECS, positive margins, pT3 or pT4 primary, N2 or N3 nodal disease, or nodal disease in levels IV or V [21]. For a patient with N1, N2a-b, or N3 disease, regardless of T stage, the surgical management arm includes excision of the primary tumor with ipsilateral or bilateral ND as indicated while, for any N2c disease, bilateral ND is mandatory [66]. Again, the presence of adverse risk factors intraoperatively is the key factor in the recommendation for adjuvant therapy in this algorithm [21]. While the NCCN guidelines provide a helpful framework for surgical management of OPSCC, they do not provide recommendations for extent of resection for either the cN0 versus cN+ neck. 

For the cN+ neck, dissection depends primarily on extent of disease. For patients presenting with gross involvement of nonlymphatic structures, including the IJV, SAN, or SCM, or for select cases of bulky, hypomobile nodal disease, a RND is the procedure of choice [7, 67]. Most institutions will offer MRND for multilevel or cN+ disease without evidence of involvement of these structures [7]. At our institution, a MRND is the procedure of choice for N+ disease. Recently, there has been some research into the use of SND for some cN+ patients. Andersen et al. and Spiro et al. have advocated SND for cN+ necks and reported a regional failure rate of 5.7% [66, 68], which is consistent with regional failure rates following RND and MRND [7]. In addition, as the risk for disease in level V is relatively low for OPSCC, many authors recommend preservation of level V, especially for cN1-N2a disease, in order to limit dissection of level V and thus reduce postoperative morbidity associated with shoulder dysfunction [67].

In the cN0 neck, it is important to remember that the consequences of undertreatment are significant, and recurrence or residual disease in an untreated cN0 neck results in a poor prognosis [2]. Weiss et al. performed a computer-assisted mathematical analysis of the decisions and associated outcomes involved in treating the cN0 neck, and concluded that the benefits of prophylactic treatment of the neck outweighed costs only when the risk for occult metastases is greater than 20% [4]. Because 15–30% of patients staged cN0 will develop nodal metastases regardless of OPSCC subsite or T stage, most authors recommend regional nodal therapy for all OPSCC primaries [14]. Currently, the SND is the procedure of choice for the cN0 neck [22]; however, the extent of resection is controversial. Recommendations vary from lateral ND (levels I–IV) to a supraomohyoid ND (levels I–III) [68–70]. Lim et al. reviewed 104 NDs to determine both the distributions of cervical lymph node metastases in OPSCC as well as the therapeutic implications for the N0 neck and recommended that elective ND should include levels II–IV instead of the more traditional levels I–III [22]. They based this recommendation on the finding that of 68 patients, who underwent therapeutic ipsilateral ND, 37% had disease in level IV while only 10% had disease in level I [22]. Shah evaluated 1119 RND specimen and found that OPSCC predominantly spreads along the jugular lymphatic chain (Levels II, III, and IV), which also supports the use of SND (level II–IV) [62]. The decision to dissect level IIB depends on risk of involvement and is controversial as described above. In 2004, Coskun et al. advocated the use of the “susper-selective ND” for some cN0 patients with OPSCC, which avoids injury to the SAN by preserving level IIB nodes [71]. At our institution, patients undergoing surgical management for an N0 neck, regardless of T stage, will most often receive a SND (level II–IV, including IIB). The choice to dissect level I depends on several factors, including the risk of involvement (higher for anterior soft palate lesions) [7], as well as the preference of the surgeon. 

An important consideration when designing an ND for OPSCC is the status of the retropharyngeal lymph nodes. This basin is not typically addressed for either the cN0 or cN+ neck, as seen above. However, RP node involvement is not uncommon, especially in posterior pharyngeal wall SCC, and can be associated with poor prognosis [12, 72]. Byers et al. reported a 4% incidence of RP nodal metastases in 45 patients staged cN0 with pharyngeal wall OPSCC [73], while Ballantyne reported an incidence as high has 44% for 34 patients with similar primary tumors [12]. Hasegawa and Matsuura concluded that since the diagnosis of RP nodes is difficult and OPSCC, especially that of the posterior pharyngeal wall, drains to the RP nodes, management of these nodes is critical [13]. Therefore, the consideration of an extended ND to include this lymphatic basin should be seriously considered when managing both cN0 and cN+ disease.

## 6. Complications

While we have discussed the oncologic benefits and morbidity associated with ND for OPSCC, one cannot ignore the acute complications of surgical intervention. The most dramatic, and often lethal, acute complication is the carotid artery rupture which is often associated with poor coverage of vasculature, malnutrition, diabetes, infection, previous radiation or chemoradiation therapy, and resultant fistula or flap breakdown [7]. Chyle leak, as a result of level IV dissection, may occur in treatment of OPSCC and occurs in up to 2% of patients [7, 74]. Additional complications such as facial or cerebral edema associated with synchronous bilateral NDs and IJV ligation, blindness associated with embolus or hypoperfusion, air embolus from IJV transection, orocutaneous fistula have all been reported for ND and should be considered when individualizing management options [7]. These risks increase with a history of radiation, chemoradiation, failed surgical therapy, and the presence of multilevel bulky disease [7].

## 7. Future Direction/Conclusions

In recent years, the use of radiation therapy and chemoradiation (CRT) therapy has been used with increasing frequency as the primary treatment modality for OPSCC tumors. This leads Kim et al. to hypothesize that there would be a decrease in the number of NDs performed nationally for primary HNSCC [75]. However, they found that from 2000 to 2006 there was a slight increase in the point estimates for ND performed for OPSCC from 2,420 to 2,696, though this increase was not significant [75]. This may be due to the decision by many surgeons to perform planned ND either prior to or following CRT or by the use of surgical intervention alone for small primary tumors cN+ or high-risk cN0 disease. Based on national trends such as these and the progressive specialization of ND based on primary site, surgical management of the neck remains an important and effective tool and should be considered in the treatment of the neck in OPSCC. 

In an effort to target specific high-risk nodal groups and decrease the percentage of patients undergoing ND for pN0 disease, future study into functional and antibody-mediated or tumor-directed imaging will be important. In addition, advancements must be made in molecular studies which will likely facilitate more effective real time SLN-directed neck dissections [2, 37]. Finally, surgical techniques which minimize morbidity without compromising oncologic safety, such as endoscopic or robotic techniques, will likely drive even more selective surgical management of the neck in OPSCC [76].

## Figures and Tables

**Table 1:** Frequency nodal level involvement in OPSCC based on ipsilateral versus contralateral neck evaluation (A), as well as clinical nodal status (B).

A. Study	Site	Level I (A/B)		Level II (A/B)*		Level III		Level IV		Level V (A/B)*	
		Ipsi (%)	Contra (%)	Ipsi (%)	Contra (%)	Ipsi (%)	Contra (%)	Ipsi (%)	Contra (%)	Ipsi (%)	Contra (%)
Grégoire and Lee [19]	OPSCC	13	2	82	24	23	5	9	2	13	3
Lindberg [8]	Soft Palate	(1.3/ 2.5)	(1.3/1.3)	37.5	12.5	11.3	2.5	2.5	0	0	1.3
	Tonsillar Fossa	(0.7/1.4)	(0/2.1)	73.6	10	17.9	6.4	10	1.4	10	3.6
	BOT	(1.1/5.4)	(0.5/0)	68.6	24.3	30.8	54.1	7	2.2	8.6	2.2
	Oropharyngeal Walls	(1.3/3.4)	(0/0)	52.3	14.1	20.8	3.4	4.7	2	9.4	4
Lim et al. [22]	OPSCC			83	57	45	50				

B. Study		Level I		Level II*		Level III		Level IV		Level V*	
		cN0 (%)	cN+ (%)	cN0 (%)	cN+ (%)	cN0 (%)	cN+ (%)	cN0 (%)	cN+ (%)	cN0 (%)	cN+ (%)

MSK**	OPSCC	2	15	25	75	19	42	8	27	2	9
Lim et al. [22]	OPSCC	0	9.9					3	35		

Ipsi: ipsilateral neck; Contra: contralateral neck; cN0: clinically negative neck; cN+: clinically positive neck; OPSCC: oropharyngeal squamous cell carcinoma; BOT: base of tongue.

*The importance of level IIB and V in OPSCC is discussed in the text under the subheading “Sequelae.”

**MSK: data from the Head and Neck Department at Memorial Sloan-Kettering Cancer Center as presented by Grégoire and Lee [19].

**Table 2:** Nodal staging of OPSCC based on the American Joint Commission on Cancer [20].

Stage	Nodal involvement
NX	The neck cannot or was not assessed.
N0	No nodal metastases; neck was evaluated.
N1	Single node, ipsilateral to primary tumor; ≤3 cm.
N2a	Single node, ipsilateral to primary tumor; 3–6 cm.
N2b	Multiple nodes, ipsilateral to primary tumor; ≤6 cm.
N2c	Single or multiple node; contralateral neck involved; all ≤6 cm.
N3	One or more nodes > 6 cm regardless of multiplicity or laterality.

**Table 3:** Neck dissection definitions based on lymphatic and nonlymphatic structures resected.

Neck dissection definition	Nonlymphatic structure			Lymphatic nodal level						Additional*
	SAN	IJV	SCM	I	II	III	IV	V	VI	
Radical	x	x	x	x	x	x	x	x		
MRND	±	±	±	x	x	x	x	x		
MRND Type I		x	x	x	x	x	x	x		
MRND Type II			x	x	x	x	x	x		
MRND Type III				x	x	x	x	x		
Complete				x	x	x	x	x		
Selective				±	±	±	±	±		
Supraomohyoid				x	x	x				
Lateral				x	x	x	x			
Posterolateral					x	x	x	x		
Anterior/central									x	
Extended	±	±	±	±	±	±	±	±	±	x

SAN: spinal accessory nerve; IJV: internal jugular vein; SCM: sternocleidomastoid muscle; I–VI: nodal levels I–VI; MRND: modified radical neck dissection.

*Additional: removal of an additional lymph node level/group or nonlymphatic structure not included in a RND. See text for examples.
